# [Expression of Concern] Myricetin inhibits migration and invasion of hepatocellular carcinoma MHCC97H cell line by inhibiting the EMT process

**DOI:** 10.3892/ol.2026.15727

**Published:** 2026-06-24

**Authors:** Hongxin Ma, Lei Zhu, Jingna Ren, Benlong Rao, Maomao Sha, Yi Kuang, Weigan Shen, Zhengxin Xu

Oncol Lett 18: 6614–6620, 2019; DOI: 10.3892/ol.2019.10998

Following the publication of the above paper, it has been drawn to the Editor's attention by a concerned reader that, regarding the cell invasion and migration assay data shown in Fig. 2B on p. 6617, two pairs of data panels, namely the Invasion, 0 μM and the Migration, 50 μM panels, and the Invasion, 0 μM and Migration, 100 μM panels, respectively, contained overlapping sections, suggesting that these data panels, which were intended to show the results of differently performed experiments, had been derived from the same original source. A subsequent investigation of the data in this paper undertaken by the Editorial Office also revealed that the Invasion, 0 μM and 50 μM panels contained a small overlapping section, suggesting that this third pair of data panels in this figure part were also derived from a common source.

After having contacted the authors about this issue, they have replied to the Editorial Office acknowledging these issues, although regrettably they are unable to repeat the relevant experiments to resolve these anomalies. Owing to the fact that the Editorial Office has received this information concerning potential issues surrounding the scientific integrity of this paper, we are issuing an Expression of Concern to notify readers of these potential problems.

